# Effect of mechanical properties of monofilament twines on the catch efficiency of biodegradable gillnets

**DOI:** 10.1371/journal.pone.0234224

**Published:** 2020-09-24

**Authors:** Eduardo Grimaldo, Bent Herrmann, Nadine Jacques, Jørgen Vollstad, Biao Su

**Affiliations:** 1 Department of Fisheries Technology, SINTEF Ocean, Trondheim, Norway; 2 Department of Fisheries Technology, SINTEF Ocean, Hirtshals, Denmark; 3 The Arctic University of Norway, UiT, Tromsø, Norway; 4 Department of Aquaculture Technology, SINTEF Ocean, Trondheim, Norway; Tanzania Fisheries Research Institute, UNITED REPUBLIC OF TANZANIA

## Abstract

Gillnets made of the biodegradable resin polybutylene succinate co-adipate-co-terephthalate were tested under commercial fishing conditions to compare their fishing performance with that of conventional nylon polyamide (PA) gillnets. Both types of gillnets were made of 0.55 mm Ø monofilaments. However, since the biodegradable nets are weaker than nylon PA nets when using the same monofilament diameter, we also used biodegradable nets made of 0.60 mm Ø monofilament that had a similar tensile strength to the 0.55 mm Ø nylon PA nets. The relative catch efficiency of the different gillnet types was evaluated over the 2018 autumn fishing season for saithe and cod in northern Norway. For cod, both biodegradable gillnets (0.55 and 0.60 mm) had a significantly lower catch efficiency compared to the traditional nylon PA net (0.55 mm) with estimated catch efficiencies of 62.38% (CI: 50.55–74.04) and 54.96% (CI: 35.42–73.52) compared with the nylon PA net, respectively. Similarly for saithe, both biodegradable gillnets (0.55 and 0.60 mm) had a lower estimated catch efficiency compared to the traditional nylon PA net (0.55 mm) with estimated catch efficiencies of 83.40% (71.34–94.86) and 83.87% (66.36–104.92), compared with the nylon PA net, respectively. Tensile strength does not explain the differences in catch efficiency between the two gillnet types, since increasing the twine diameter of the biodegradable gillnets (to match the strength of nylon PA gillnets) did not yield similar catch efficiencies. However, the elasticity and stiffness of the materials may be responsible for the differences in catch efficiency between the nylon PA and biodegradable gillnets.

## Introduction

Globally, gillnets are among the most commonly used fishing gears in developing and industrialized countries [1]. In Norway, 26% and 16% of the total national allowable quota for Northeast Atlantic cod (*Gadus morhua*) and saithe (*Pollachius virens*), which in 2019 was 385,000 and 203,368 tonnes respectively, were caught with gillnets [2]. The Norwegian coastal fleet (with vessels shorter than 28 m) is responsible for approximately 99% of the gillnet landings of Northeast Atlantic cod. In 2019, the coastal fleet consisted of 5,978 vessels, with 81% of them being smaller than 14.9 m [3]. The coastal fleet is also responsible for a large number of gillnets lost every year, causing environmental problems such as ghost fishing and marine litter. Deshpande et al. [4] provided annual loss rates of the six types of fishing gears used in Norwegian waters, and gillnets were the primary source of derelict gear. Although fisheries authorities lack a complete overview of the amount of lost or derelict gillnets, estimates from the Norwegian Environment Agency [5] suggest that 13,941 gillnets are lost each year.

The impacts of derelict gillnets include continued catching of target and non-target species (commonly known as ghost fishing), alterations to the benthic environment, marine plastic pollution, navigational hazards, beach debris/litter, introduction of synthetic material into the marine food web, and costs related to clean-up operations [6]. The impact of derelict gillnets on the environment has been exacerbated by the introduction of non-biodegradable materials, primarily nylon polyamide (PA), which are generally more persistent in the environment than natural materials. Ghost fishing also represents an unregistered amount of fishing mortality [7], which undermines the use of the population analysis models for maximum sustainable yield management and the ecosystem management approach. There have been extensive efforts to assess the magnitude of derelict gillnets [8, 9], and in the last decade many studies have focussed on developing methods to reduce the effects of derelict gear. Some specific measures to address the problem include gear marking, onshore collection/reception and/or payment for old/retrieved gear, reduced fishing effort, use of biodegradable nets, and gear recovery programs for gear disposal and recycling [9].

Norway is one of the countries that has a program to systematically retrieve lost gears from areas with the highest fishing intensity. Between 1983 and 2017, the Norwegian Directorate of Fisheries retrieved 20,450 lost gillnets and a large amount of other fishing gear (e.g., ropes, pots, trawls), which contained variable amounts of marine resources that had been caught in the lost gear. In 2017, just 815 of the 13,941 estimated lost gillnets were retrieved [5, 10]. Due to the low recovery rate of lost fishing gears and the low on-land disposal rate of plastics from the fishing industry [10], recent research has focused on developing biodegradable plastic materials for fishing gear, i.e. gillnets, to try to reduce the negative effects of derelict fishing gear.

Biodegradable plastic is a plastic that maintains similar properties as a conventional plastic during use, but that can be completely degraded by naturally occurring microorganisms such as bacteria, fungi, and algae when disposed of in the environment [11]. The most investigated biodegradable plastics in fishing equipment and other marine applications, i.e., aquaculture, are polybutylene succinate, polybutylene adipate co-terephthalate, and polybutylene succinate co-adipate-co-terephthalate [12–20]. Commercial fishing products made of these materials are available in some countries, such as South Korea. Various microorganisms are known to degrade biodegradable plastics at different rates, for example, the microorganisms present in the Arctic have a high capacity for biodegradation [21]. Additionally, there are reports that the degradation of polycaprolactone and poly(3-hydroxybutyrate-co-3-hydroxyvalerate) fibres occurs at a faster rate than that of polybutylene succinate fibres in deep seawater [22]. Biodegradable fishing nets have thermal, mechanical, and physical properties that are similar to those of traditional products made of nylon PA, polyester, polyethylene, and polypropylene [12, 13, 17].

Biodegradable fishing gears have been studied in South Korea and Norway as an alternative to reduce the negative impact of derelict gear on the marine environment. In South Korea, these gears have been tested in 13 different fisheries, including gillnetting and potting for roundfish, flatfish, shrimps, octopus, crabs, and eels [12–17, 23–25]. The results showed that in some cases the fishing efficiency of these gears is similar to that of gears made of PA, polyethylene, and polypropylene. In Norway, biodegradable PBSAT gillnets have shown a consistently lower catch efficiency than nylon PA gillnets, and this difference has been mainly attributed to the fact that biodegradable gillnets are made with 11–16% weaker monofilaments than nylon PA monofilaments of the same diameter [18–20, 26]. The aim of the present study is to assess the effect of twine thickness and tensile strength on the catch efficiency of biodegradable PBSAT gillnets. Our main hypothesis is that by increasing the monofilament diameter of the biodegradable gillnets to match the tensile strength of nylon PA monofilaments, the catch efficiency of the biodegradable gillnets will yield a similar catch efficiency to nylon PA gillnets. We designed the experiments to answer the following research questions:

- Can biodegradable and nylon PA gillnets made of monofilaments with similar tensile strength (although different monofilament diameter) yield similar catch efficiencies?
- Is tensile strength the mechanical property responsible for the difference in catch efficiency between biodegradable and nylon PA gillnets?
- Is catch efficiency positively or negatively correlated to monofilament diameter in biodegradable gillnets?

## Materials and methods

### Ethics statement

This study did not involve endangered or protected species. Experimental fishing was conducted on board a commercial fishing vessel and no permit was required to conduct the study on board. No information on animal welfare, or on steps taken to mitigate fish suffering and methods of sacrifice is provided, since the animals were not exposed to any additional stress other than that involved in commercial fishing practices.

### Experimental setup

Sea trials were conducted on board the coastal gillnet vessel "MS Karoline" (10.9 m total length) during the autumn fishing season for saithe and cod in northern Norway. The fishing grounds chosen for the sea trials were located off the coast of Troms (northern Norway) between 70°21’–70°22’N and 19°39’–19°42’E, which is a common fishing area for coastal vessels from Troms.

We used gillnets with 130 mm nominal mesh size because it is the most commonly used mesh size used by the fleet during the autum season. The monofilament thickness was 0.55 and 0.60 mm in the biodegradable gillnets and 0.55 mm in the nylon PA gillnets. Since biodegradable monofilaments are 11–16% weaker than nylon PA monofilaments of the same diameter [18–20, 26], we increased the diameter of the biodegradable monofilament from 0.55 to 0.60 mm to compensate for the difference in tensile strength. Assumimg a linear relationship between monofilament thickness and breaking strength an increase from 0.55 to 0.60 mm would be equivalent to a 9% increase in strength. We also consider a second order relationship (monofilaments cross area), and according to this relationship and increment of the monofilament diameter from 0.55 mm to 0.60 mm would be equivalent to approximately 18% increase in strength. Actual measurements of the mesh openings (four rows of 20 meshes each) were taken with a Vernier calliper without applying tension to the meshes. They showed that the mean mesh openings of 0.55 mm nylon PA gillnets and 0.55 mm and 0.60 mm biodegradable gillnets were 131.6 ± 0.72 mm, 131.5 ± 1.0 mm and 132.5 ± 0.8 mm, respectively. Each gillnet sheet was 50 meshes high by 275 meshes long (approx. 55 m stretched length). Each assembled gillnet was approximately 27.5 m long and had a hanging ratio of 0.5. To provide buoyancy, each gillnet sheet was fixed to 26 mm diameter SCANFLYT-800 floatlines (made of braided polypropylene rope with a single core of polyurethane floating elements inside) with a buoyancy of 150 g m^–1^. To provide weight, they were each attached to a 16 mm diameter DANLINE leadline (made of polypropylene rope with a lead core) with a weight of 360 g m^–1^.

Two sets of gillnets were used in the experiments. In one set we compared 0.55 mm biodegradable gillnets with 0.55 mm nylon PA gillnets. In the other set we compared 0.60 mm biodegradable gillnets with 0.55 mm nylon PA gillnets. Each set consisted of 16 gillnets, with eight biodegradable gillnets (B) and eight nylon PA gillnets (N). The gillnets were arranged in such a way that they provided information for paired comparison, nylon PA versus biodegradable gillnet, accounting for spatial and temporal variation in the availability of cod. With individual sets being the basic unit for the paired analysis [19], it was important that the biodegradable and nylon PA gillnets were approximately exposed to the same spatial variability in fish availability within each gillnet set. This could in principle be achieved by alternating between the two types of nets after each net sheet as follows: B-N-B-N-B-N-B-N-B-N-B-N-B-N-B-N. However, for ease of on board recording of fish in relation to the type of net in which it was caught, the alternation in net types was only applied after every second net sheet. Therefore, to make conditions as equal as possible between net types, set 1 was arranged as N-B-B-N-N-B-B-N-N-B-B-N-N-B-B-N and set 2 as B-N-N-B-B-N-N-B-B-N-N-B-B-N-N-B. The distance between gillnets was approximately 1 meter.

A total of 22 gillnet deployments were carried out throughout the experimental period. Scientists on board the "MS Karoline" sorted out the catch by type of gillnet and measured the total lengths (to the nearest cm) of all fish caught in 21 deployments. Data from one deployment (on November 26^th^) was lost.

### Data analysis

#### Modelling catch efficiency

We used the statistical analysis software SELNET [27, 28] to analyze the catch data and conduct length-dependent catch comparison and catch ratio analyses. Using the catch information (numbers and sizes of cod or saithe in each gillnet set deployment), we wanted to determine whether there was a significant difference in the catch efficiency averaged over deployments between the nylon PA gillnet and the biodegradable gillnet. We also wanted to determine if a potential difference between the gillnet types could be related to the size of the cod or saithe. The analysis was conducted separately for each species (cod and saithe) and each biodegradable gillnet (0.55 mm and 0.60 mm) following the procedure described below.

To assess the relative length-dependent catch efficiency effect of changing from nylon PA gillnet to a biodegradable gillnet, we used the method described by Herrmann et al. [29] and compared the catch data for the two net types. This method models the length-dependent catch comparison rate (*CC*_*l*_) summed over gillnet set deployments:

$${CC}_{l}=\frac{\sum_{j=1}^{m}\left\{{nt}_{lj}\right\}}{\sum_{j=1}^{m}\left\{{nt}_{lj}+{nc}_{lj}\right\}}$$ (1)

where *nc*_*lj*_ and *nt*_*lj*_ are the numbers of fish caught in each length class *l* for the nylon PA gillnet (control) and the biodegradable gillnet (treatment) in deployment *j* of a gillnet set (first or second set). *m* is the number of deployments carried out with one of the two sets. The summation in Eq 1 implies the results are obtained pooling all sets and deployments. The functional form for the catch comparison rate *CC(l*,***v****)* was obtained using maximum likelihood estimation by minimizing the following expression:

$$-\sum_{l}\left\{\sum_{j=1}^{m}\left\{{nt}_{lj}\times ln(CC(l,v))+{nc}_{lj}\times ln(1.0-CC(l,v))\right\}\right\}$$ (2)

where ***v*** is a vector of the parameters describing the catch comparison curve defined by *CC(l*,***v****)*. The outer summation in the equation is the summation over length classes *l*. When the catch efficiency of the biodegradable gillnet and nylon PA gillnet is similar, the expected value for the summed catch comparison rate would be 0.5. Therefore, this baseline can be applied to judge whether or not there is a difference in catch efficiency between the two gillnet types. The experimental *CCl* was modelled by the function *CC(l*,***v****)* using the following equation:

$$CC(l,v)=\frac{exp(f(l,v_{0},\ldots ,v_{k}))}{1+exp(f(l,v_{0},\ldots ,v_{k}))}$$ (3)

where *f* is a polynomial of order *k* with coefficients *v*_*0*_ to *v*_*k*_. The values of the parameters ***v*** describing *CC(l*,***v****)* were estimated by minimizing Eq (2), which was equivalent to maximizing the likelihood for the observed catch data. We considered *f* of up to an order of 4 with parameters *v*_*0*_, *v*_*1*_, *v*_*2*_, *v*_*3*_, and *v*_*4*_. Leaving out one or more of the parameters *v*_*0*_…*v*_*4*_ led to 31 additional models that were also considered as potential models for the catch comparison *CC(l*,***v****)*. Among these models, estimations of the catch comparison rate were made using multi-model inference to obtain a combined model [29, 30]. Specifically, the models were ranked and weighed in the estimation according to their AICc values [29]. The AICc is calculated as the AIC [31], but it includes a correction for finite sample sizes in the data. Models that resulted in AICc values within +10 of the value of the model with lowest AICc value were considered for the estimation of *CC(l*,***v****)* following the procedure described in Herrmann et al. [32] and in Katsanevakis [33].

The ability of the combined model to describe the experimental data was evaluated based on the p-value. The p-value, which was calculated based on the model deviance and the degrees of freedom, should not be < 0.05 for the combined model to describe the experimental data sufficiently well, except for cases where the data are subject to over-dispersion [29, 34]. Based on *CC(l*,***v****)* we obtained the relative catch efficiency (also named catch ratio) *CR(l*,***v****)* between the two gillnet types using the following relationship:

$$CR(l,v)=\frac{CC(l,v)}{\left(1-CC(l,v)\right)}$$ (4)

The catch ratio is a value that represents the relationship between catch efficiency of the biodegradable gillnet and that of the nylon PA gillnet. Thus, if the catch efficiency of both gillnets is equal, *CR(l*,***v****)* should always be 1.0. *CR(l*,***v****)* = 1.5 would mean that the biodegradable gillnet is catching 50% more cod of length l than the nylon PA gillnet. In contrast, *CR(l*,***v****)* = 0.8 would mean that the biodegradable gillnet is only catching 80% of the cod of length l that the nylon PA gillnet is catching.

The confidence limits for the catch comparison curve and catch ratio curve were estimated using a double bootstrapping method [29]. This bootstrapping method accounts for between-set variability (the uncertainty in the estimation resulting from set deployment variation of catch efficiency in the gillnets and in the availability of fish) as well as within-set variability (uncertainty about the size structure of the catch for the individual deployments). However, contrary to the double bootstrapping method [29], the outer bootstrapping loop in the current study, which accounts for between deployment variation, was performed as a paired analysis. By multi-model inference in each bootstrap iteration, the method also accounted for the uncertainty due to uncertainty in model selection. We performed 1,000 bootstrap repetitions and calculated the Efron 95% [35] confidence limits. To identify sizes of fish with significant differences in catch efficiency, we checked for length classes in which the 95% confidence limits for the catch ratio curve did not contain 1.0.

Additonally, a length-integrated average value for the catch ratio was estimated directly from the experimental catch data using the following equation:

$${CR}_{average}=\frac{\sum_{l}\sum_{j=1}^{m}\left\{{nt}_{lj}\right\}}{\sum_{l}\sum_{j=1}^{m}\left\{{nc}_{lj}\right\}}$$ (5)

where the outer summation covers the length classes in the catch during the experimental fishing period.

Finally, to investigate the effect that the accumulated number of times the gillnets were deployed (*DN*) had on the length-integrated catch ratio (*CR*_*average*_), Eq (5) was calculated for individual deployment sets without the summation over gillnet sets. This led to a dataset consisting of pair values for the number of times the gillnets were deployed and the corresponding values for *CR*_*average*_. Based on this dataset, we tested if the value for *CR*_*average*_ changed linearly with the number of deployment times using the following equation:

$${CR}_{average}(DN)=\alpha \times DN+\beta$$ (6)

The last part of the analysis using model (6) was conducted using the linear model function (lm) in the statistical package R (version 2.15.2; www.r-project.org).

#### Assessing the catch ratio of the two biodegradable gillnet designs

Because the same nylon gillnet design was used as a baseline in the asssessment of the catch ratio curves for both the 0.55 and 0.60 mm biodegradable gillnet, it was possible to indirectly assess the catch ratio curve between the two biodegradable gillnets. This was performed by calculating the ratio between the catch ratio curves obtained from the two catch ratio curves against the nylon net using the following equation:

$${CR(l,v)}_{0.60/0.55}=\frac{{CR(l,v)}_{0.60}}{{CR(l,v)}_{0.55}}$$ (7)

The 95% confidence intervals for *CR*(*l,**v***)_0.60/0.55_ were obtained based on the two bootstrap populations of results (1000 bootstrap repetitions in each) from each CR curve estimated for the 0.55 and 0.60 mm biodegradable gillnets against the nylon net. Since both bootstrap populations were obtained independently and the sampling to obtain those populations of results was performed randomly and independently, a new population of results with 1000 bootstrap iterations was created for *CR*(*l,**v***)_0.60/0.55_ following [36, 37]:

$${CR(l,v)}_{{0.60/0.55}_{i}}=\frac{{CR(l,v)}_{0.60_{i}}}{{CR(l,v)}_{0.55_{i}}}\;,\;i\in [1,\ldots ,1000]$$ (8)

Where *i* represents the bootstrap repetion index. Based on this new population the Efron 95% confidence bands for *CR*(*l,**v***)_0.60/0.55_ were obtained.

#### Assessment of mechanical properties

**Fig 1:**
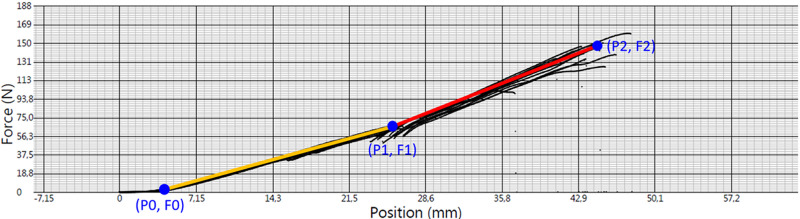
Elasticity: Estimation of *k*_*1*_ and *k*_*2*_ from force–displacement curve. *F* is the applied force and *P* is the displacement (amount of elongation).

Tensile testing were carried out on all the biodegradable and nylon PA gillnets used in the fishing experiments using a H10KT universal tensile testing machine (Tinius Olsen TMC, PA, USA) equipped with a load cell with 5000 Newton (N) rated force. The tests were performed in wet conditions on samples collected before (new nets) and after the experimental fishing (used nets) (at least 40 replicates for each case) according to the ISO 1806:2002 [38]. We estimated the mean tensile strength, elongation at break and the elasticity of the samples. Tensile strength, defined as the stress needed to break the sample, is given in kg. Elongation at break, defined as the length of the sample after it had stretched to the point when it breaks, is given as a percentage relative to the initial mesh size. Elasticity is a measurement of the resistance of an object or substance to being deformed elastically when a force is applied to it. Elasticity is a property of a material representing its ability to completely regain its original shape and size after removal of applied load. Stiffness is a physical quantity that represents the magnitude of force required to cause unit deformation. The outputs from tensile testing were force-displacement curves which are described by the followeing equation:

F=−k×ΔP (9)

where Δ*P* is the amount of deformation (displacement in mm) produced by the force *F*, and *k* is a measure for the elasticity that depends on the shape and composition of the object and the direction of the force. The bigger *k* is, the stiffer the material is. Because the relationship between the force and the displacement is not linear, we estimated two elasticities from the slopes of the force–displacement curve in the elastic deformation region (Fig 1). For low deformations we used *k*_*1*_ and for larger deformations we used *k*_*2*_ according to the following equations:

$$k_{1}=\frac{\left(F_{1}-F_{0}\right)}{\left(P_{1}-P_{0}\right)};\;k_{2}=\frac{\left(F_{2}-F_{1}\right)}{\left(P_{2}-P_{1}\right)}$$ (10)

where *P*_*0*_ is the displacement position at *F*_*0*_, *P*_*1*_ is the displacement position at 50% of the breaking point that corresponds to the *F*_*1*_ and *P*_*2*_ and *F*_*2*_ is the dispalcement and force at the breaking point.

Force-elongation curves were obtained from tensile testing for all types of gillnets, new and used. For each replicate, the tensile strength was determined as the peak of the force-elongation curve, and the corresponding elongation was taken as the elongation at break. For a set of samples, the tensile strength was determined as the average of all replicates, and polynomial fitting was performed to determine the average force-elongation curve. The ratio of force-elongation is elasticity-stiffness, but only the force defines the strength of the material. Strength measures how much stress the material can handle before permanent deformation or fracture occurs, whereas stiffness measures the resistance to elastic deformation.

Force-elongation curves of new and used nylon PA and biodegradable gillnets from experiments carried out in 2016–2019 [18–20] are presented and used in the discussion section to support the findings of this study. These curves were estimated by following the same tensile testing procedure as described above.

## Results

Data were collected for both cod and saithe throughout the trial period. A total of 1,200 cod were caught, 780 using the nylon PA gillnet and 420 in the biodegradable gillnet (269 with the 0.55 mm and 151 with the 0.60 mm nets). A total of 1,328 saithe individuals were collected, of these, 736 were caught in the nylon PA gillnets and the remaining 592 were caught in the biodegradable gillnet (403 with the 0.55 mm and 189 with the 0.60 mm nets). Data were collected for 21 gillnet deployments for both cod and saithe, but the analysis was conducted based on deployments that had at least 10 fish in each set (Table 1). This was done in order to reduce the potential for additional uncertainty in the results and has been used successfully in previous catch comparison studies [18, 19].

**Table 1:** Catch data from all deployments for cod.

Set	Setup Nylon PA/Bionet	Setting date (dd.mm.yyyy)	Fishing time	Fishing depth (m) (min–max)	Acc. no. of deployments	No. of cod in nylon PA gillnets	No. of cod in bio gillnets	Cod length in nylon PA gillnets (min–max)	Cod length in bio gillnets (min–max)
1	0.55/0.55	07.09.2018	19h 45min	140	1	1	1	87–87	60–60
1	0.55/0.60	07.09.2018	19h 45min	120	1	0	0	0–0	0–0
2	0.55/0.55	11.09.2018	21h 45min	110	2	3	1	60–85	64–64
2	0.55/0.60	11.09.2018	22h 10min	130	2	2	3	66–76	60–101
3	0.55/0.55	31.10.2018	27h 30min	170–140	3	15	7	51–88	50–73
3	0.55/0.60	31.10.2018	26h 15min	130–110	3	1	2	80–80	61–63
4	0.55/0.55	01.11.2018	22h 40min	180–160	4	6	2	59–69	60–64
4	0.55/0.60	01.11.2018	24h 15min	110–130	4	1	2	65–65	50–67
5	0.55/0.55	02.11.2018	22h 40min	100–120	5	3	2	63–73	65–68
5	0.55/0.60	02.11.2018	23h 55min	105–125	5	2	2	63–68	60–64
6	0.55/0.55	12.11.2018	24h 50min	25–30	6	40	28	60–88	59–84
6	0.55/0.60	12.11.2018	24h 15min	50–70	6	6	3	61–81	67–73
7	0.55/0.55	13.11.2018	21h 20min	25–30	7	4	1	56–66	78–78
7	0.55/0.60	13.11.2018	21h 45min	50–70	7	4	0	60–68	59–91
8	0.55/0.55	14.11.2018	22h 00min	50–70	8	2	4	59–69	60–90
8	0.55/0.60	14.11.2018	18h 20min	50–70	8	1	3	74–74	56–83
9	0.55/0.55	27.11.2018	22h 20min	35–20	9	27	11	52–86	55–92
9	0.55/0.60	27.11.2018	23h 20min	95–45	9	11	0	55–77	0–0
10	0.55/0.55	28.11.2018	23h 20min	35–20	10	14	6	53–76	56–75
10	0.55/0.60	28.11.2018	22h 20min	50–85	10	1	2	66–66	64–69
11	0.55/0.55	29.11.2018	23h 40min	38–25	11	30	9	53–68	56–75
11	0.55/0.60	29.11.2018	26h 20min	55–45	11	12	7	50–74	56–71
12	0.55/0.55	30.11.2018	18h 05min	30–75	12	36	23	52–92	54–87
12	0.55/0.60	30.11.2018	18h 55min	45–48	12	11	13	57–98	53–84
13	0.55/0.55	01.12.2018	25h 40min	30–75	13	26	18	56–96	66–96
13	0.55/0.60	01.12.2018	26h 00min	45–48	13	24	8	51–94	67–95
14	0.55/0.55	02.12.2018	18h 05min	30–76	14	20	7	50–85	54–67
14	0.55/0.60	02.12.2018	18h 15min	45–49	14	100	12	50–92	51–95
15	0.55/0.55	03.12.2018	26h 10min	35–20	15	33	17	50–95	56–78
15	0.55/0.60	03.12.2018	28h 05min	50–85	15	16	11	51–96	58–87
16	0.55/0.55	04.12.2018	16h 00min	30–75	16	28	14	50–84	55–66
16	0.55/0.60	04.12.2018	16h 15min	45–48	16	11	6	52.92	62–96
17	0.55/0.55	06.12.2018	23h 00min	30–75	17	46	47	52.95	51–76
17	0.55/0.60	06.12.2018	23h 25min	45–48	17	50	44	55–94	50–94
18	0.55/0.55	07.12.2018	25h 20min	30–75	18	19	12	54.67	52–72
18	0.55/0.60	07.12.2018	22h 20min	45–48	18	26	4	52–95	64–85
19	0.55/0.55	08.12.2018	24h 05min	30–75	19	26	22	50–74	52–67
19	0.55/0.60	08.12.2018	27h 55min	45–48	19	15	10	56–85	55–86
20	0.55/0.55	09.12.2018	22h 50min	30–75	20	27	12	52–87	50–89
20	0.55/0.60	09.12.2018	18h 10min	45–48	20	32	9	54–92	59–87
21	0.55/0.55	10.12.2018	16h 30min	30–75	21	26	25	54–71	51–82
21	0.55/0.60	10.12.2018	16h 05min	45–48	21	22	10	55–96	51–95

The rows highlighted in grey indicate sets used in the analysis (sets containing at least 10 cod). The setups with 0.55 mm nylon PA gillnets / 0.55 or 0.60 mm biodegradable gillnets are indicated by 0.55/0.55 and 0.55/0.60.

**Fig 2:**
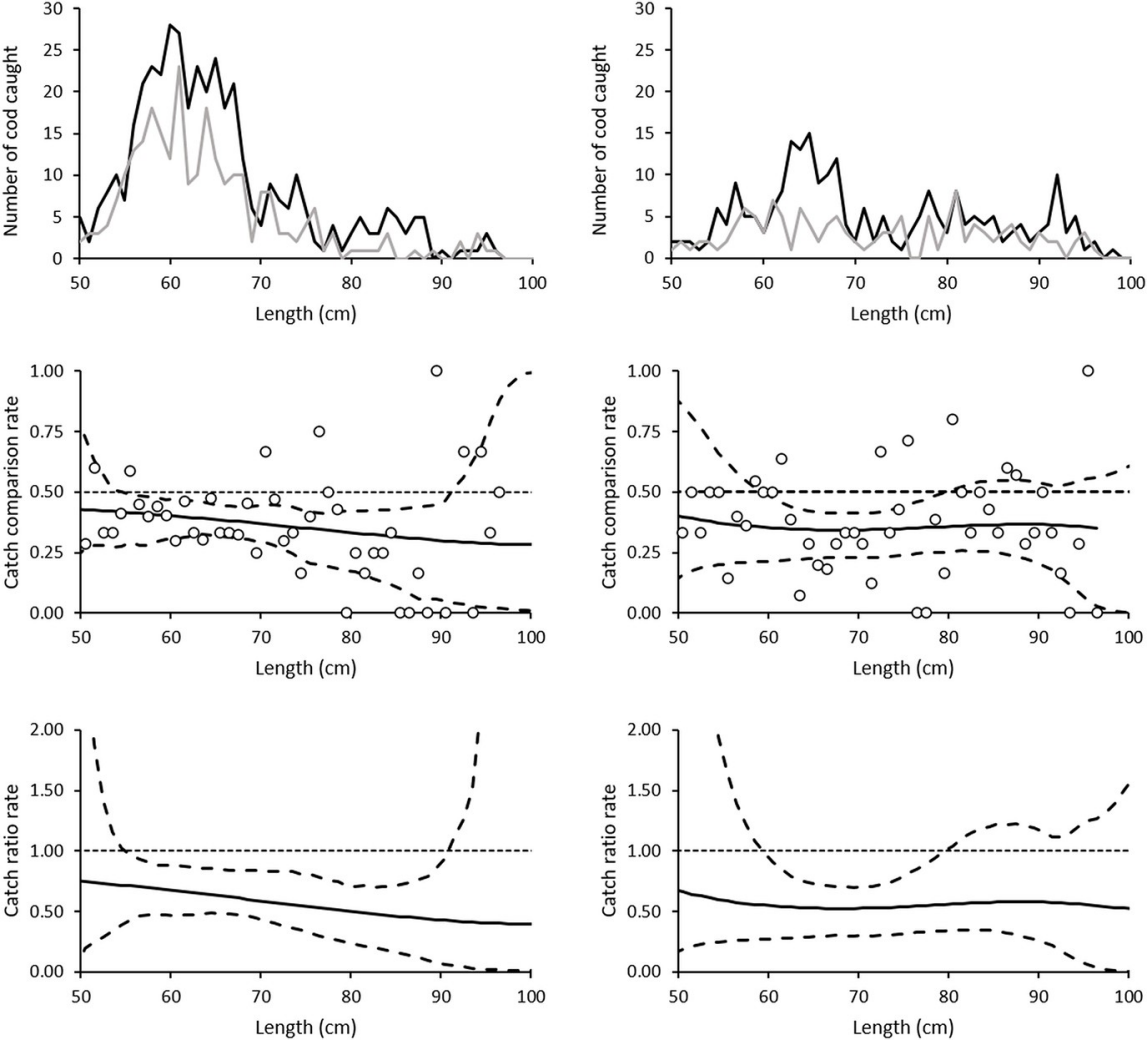
Size distribution, catch comparison rate and catch ratio rate for cod. The upper figures show the size distribution of cod caught using 0.55 mm nylon PA (black), and 0.55 mm (left) and 0.60 mm (right) biodegradable (grey) twine gillnets. The figures in the middle show the catch comparison curve for cod, with circle marks indicating the experimental rate, and the curve indicates the modelled catch comparison rate. The dotted line at 0.5 indicates the baseline where both gillnets fish the same amount. The dashed curves represent the 95% confidence interval for the estimated catch comparison curve. The lowest figure shows the estimated catch ratio curve for cod (solid line). The dotted line at 1.0 indicates the baseline where the fishing efficiency of both gillnet types is equal. The dashed curves represent the 95% confidence intervals of the estimated catch ratio curve.

For cod, this resulted in a total of 15 sets for analysis from the 0.55 mm setup and 13 from the 0.60 mm setup (Table 1). The catch was length-dependent for both types of gillnet, including fish from 50 to 101 cm, but with most of the fish in the range of 55 to 75 cm (Fig 2, S1 File). The catch efficiency of the 0.55mm biodegradable gillnets was significantly lower than that of the nylon PA gillnets for almost all cod sizes except for those below 56 cm and larger than 92 cm, while that of the 0.60 mm biodegradable gillnets was significantly lower only for sizes between 60 to 80 cm. Averaged over all length classes, both biodegradable gillnets (0.55 and 0.60 mm) had a significantly lower catch efficiency compared to the traditional nylon PA gillnet (0.55 mm) with estimated efficiencies of 62.38% (CI: 50.55–74.04) and 54.96% (CI: 35.42–73.52) respectively, meaning that the biodegradable gillnets on average caught approximately 37.62% and 45.04% fewer fish than the nylon PA gillnets (Table 2 and Fig 2).

**Table 2:** Catch rate and fit statistic results from the 0.55 and 0.60 mm biodegradable gillnets vs. the 0.55 mm nylon PA set based on valid deployments for cod.

Length (cm)	Catch ratio (%)	
	0.55 mm	0.60 mm
50	74.59 (24.39–269.67)	65.93 (24.43–410.77)
55	70.97 (46.14–96.63)	58.57 (28.60–139.11)
60	66.97 (47.25–87.92)	54.41 (29.05–94.91)
65	62.66 (47.73–84.43)	52.63 (29.65–74.56)
70	58.17 (40.29–82.65)	52.61 (30.64–70.73)
75	53.72 (29.74–80.38)	53.90 (31.20–83.56)
80	48.70 (21.37–70.54)	55.90 (33.45–106.62)
85	45.71 (13.67–72.52)	57.63 (33.27–126.53)
90	42.56 (4.97–93.69)	57.74 (28.19–116.21)
95	40.37 (1.62–320.05)	55.26 (9.76–109.90)
Average	62.38 (50.55–74.04)	54.96 (35.42–73.52)
P–value	0.2915	0.0334^'^
Deviance	45.46	60.29
DOF	41	42

Values in parentheses represent 95% confidence intervals. DOF denotes degrees of freedom.

**Fig 3:**
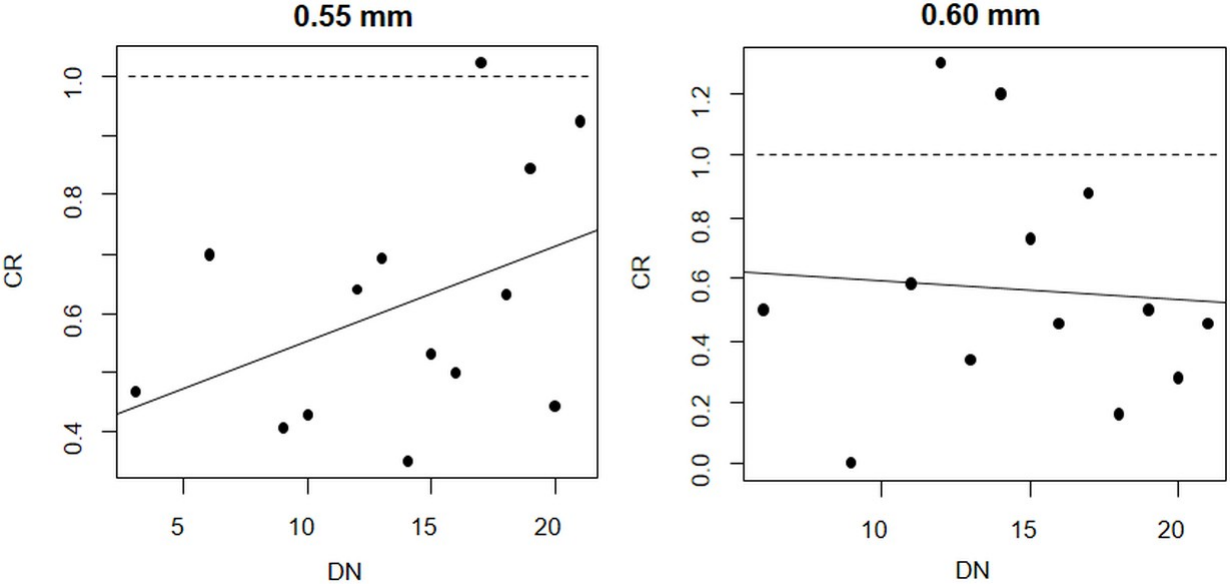
Fit of the linear model (thick solid line) testing the effect of number of times the gillnets were deployed (DN) on *CR*_*average*_ cod. At 1.0, both biodegradable and nylon PA gillnets fish equally. The circle marks represent the experimental length-integrated catch ratio (*CR*_*average*_) for individual deployments.

No clear effect of the number of times the gillnets were deployed on the relative catch efficiency between biodegradable and nylon PA gillnet was found for cod (Fig 3). For gillnets of 0.55 mm an increase was indicated while for gillnets of 0.60 mm it was a decrease. However, in neither case the effect was found to be significant as the p-value for the slope parameter α > 0.05 (Table 3).

**Table 3:** Results from linear modelling of the effect of number of times the gillnets were deployed on *CR*_*average*_ cod.

	parameter	value	Standard error	Significance (*p-*value)
	*α*	0.01584	0.01011	0.1432
0.55 mm	*β*	0.39466	0.14876	0.0211
	*R*^*2*^*-value*	0.1698		
	*α*	-0.00634	0.02564	0.809
0.60 mm	*β*	0.66086	0.39226	0.120
	*R*^*2*^*-value*	0.0055		

**Fig 4:**
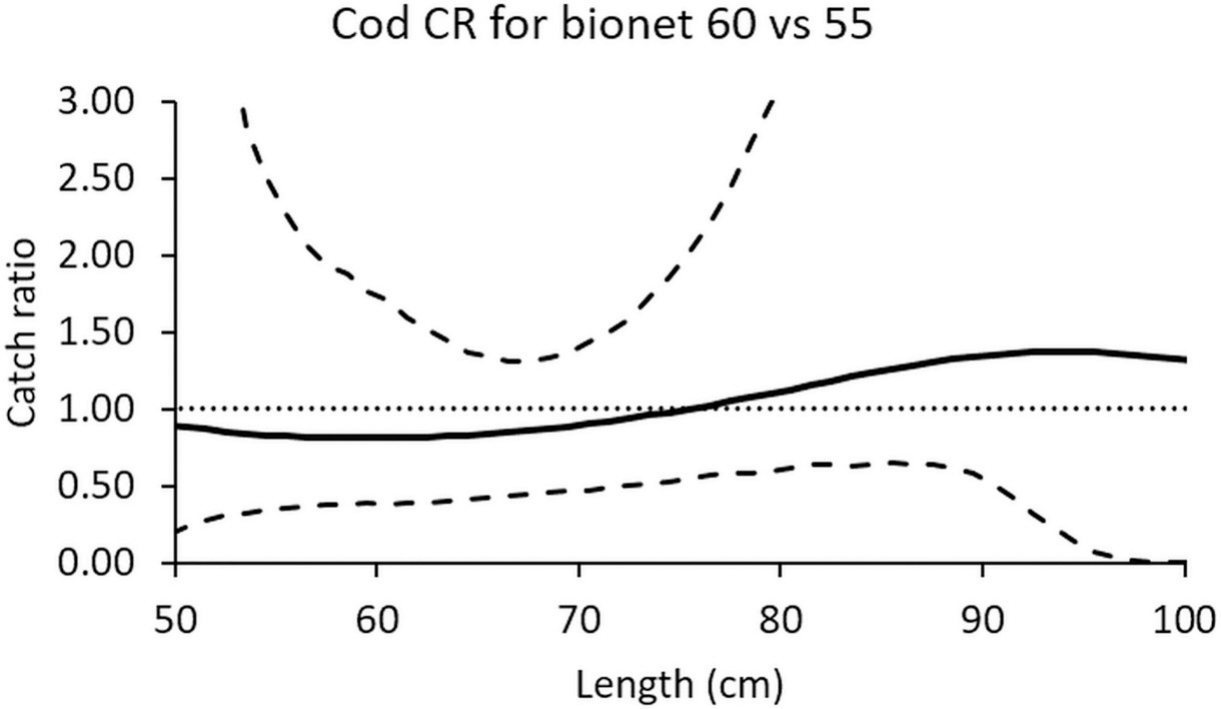
Relative catch efficiency between the two biodegradable gillnet designs for cod (solid line). The dashed curves represent the 95% confidence intervals of the estimated catch ratio curve. The dotted line at 1.0 indicates the baseline where the fishing efficiency of both gillnet types is equal.

Increasing the monofilament diameter from 0.55 mm to 0.60 mm did not have a significant effect on the catch efficiency of biodegradable gillnets. Both types of gillnets caught a similar number of cod in all length classes (Fig 4).

**Fig 5:**
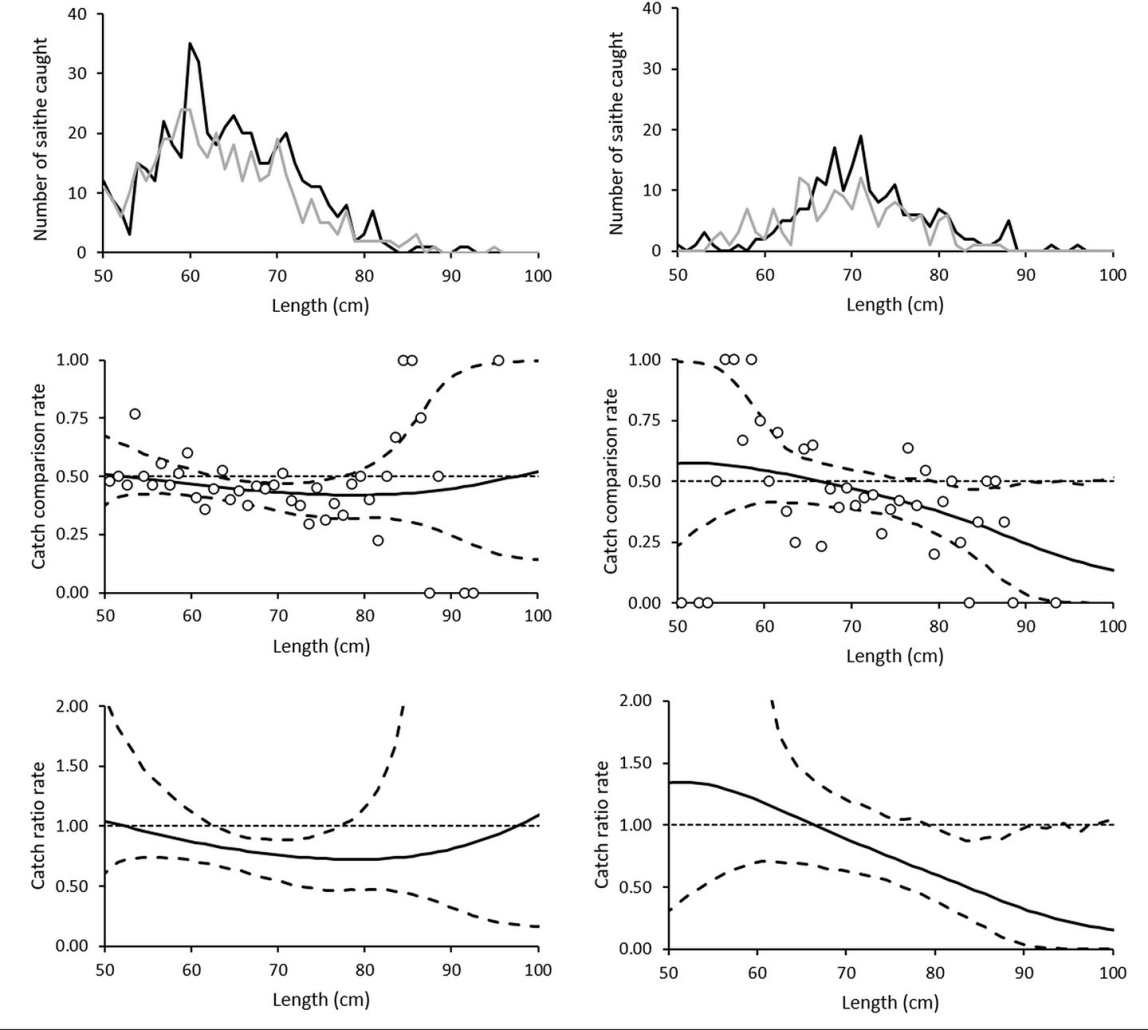
Size distribution, catch comparison rate and catch ratio rate for saithe. The upper figure shows the size distribution of saithe caught using the 0.55 mm nylon PA (black), and 0.55 mm (left) and 0.60mm (right) biodegradable (grey) twine gillnets. The figure in the middle shows the estimated catch ratio curve for saithe (solid line). The dotted line at 1.0 indicates the baseline where the fishing efficiency of both gillnet types is equal. The dashed curves represent the 95% confidence interval of the estimated catch ratio curve. The lowest figure shows the catch comparison curve for saithe, with circle marks indicating the experimental rate, and the curve indicates the modelled catch comparison rate. The dotted line at 1.0 indicates the baseline where fishing efficiency of both gillnet types is equal. The dashed curves represent the 95% confidence intervals of the estimated catch ratio curve.

For saithe, there were 15 sets for analysis of the 0.55 mm setup and 11 for the 0.60 mm setup (Table 4). The catch efficiency of the 0.55mm biodegradable gillnets was significantly lower than that of the nylon PA gillnets for sizes between 65 and 78 cm, while that of the 0.60 mm biodegradable gillnets was significantly lower for fish larger than 79 cm (Fig 5 and S2 File). Averaged over all length clases, the 0.55mm biodegradable gillnets) had a significantly 83.40% (71.34–94.86) lower catch efficiency for saithe compared to the traditional nylon 0.55 mm PA net. The average catch efficiency of the 0.60mm biodegradable gillnets was 83.87% (66.36–104.92) lower than that of the nylon PA gillnets, however the difference was not statistically significant compared with the nylon PA net, (Table 5 and Fig 5).

**Table 4:** Catch data from all deployments for saithe.

Set	Setup	Setting date	Fishing time	Fishing depth (m) (min–max)	Acc. no. of deployments	No. of saithe in nylon PA gillnets	No. of saithe in bio gillnets	Saithe length in nylon PA gillnets (min–max)	Saithe length in bio gillnets (min–max)
	Nylon PA/Bionet	(dd.mm.yyyy)							
1	0.55/0.55	07.09.2018	19h 45min	140	1	4	2	64–74	64–67
1	0.55/0.60	07.09.2018	19h 45min	120	1	0	0	0–0	0–0
2	0.55/0.55	11.09.2018	21h 45min	110	2	3	0	73–83	0–0
2	0.55/0.60	11.09.2018	22h 10min	130	2	3	2	67–70	69–73
3	0.55/0.55	31.10.2018	27h 30min	170–140	3	9	4	54–69	50–75
3	0.55/0.60	31.10.2018	26h 15min	130–110	3	3	0	50–75	0–0
4	0.55/0.55	01.11.2018	22h 40min	180–160	4	3	1	65–76	70–70
4	0.55/0.60	01.11.2018	24h 15min	110–130	4	0	1	0–0	50–50
5	0.55/0.55	02.11.2018	22h 40min	100–120	5	4	2	62–77	63–70
5	0.55/0.60	02.11.2018	23h 55min	105–125	5	5	3	61–71	59–68
6	0.55/0.55	12.11.2018	24h 50min	25–30	6	21	13	59–83	59–86
6	0.55/0.60	12.11.2018	24h 15min	50–70	6	17	8	52–87	56–77
7	0.55/0.55	13.11.2018	21h 20min	25–30	7	3	1	67–72	68–68
7	0.55/0.60	13.11.2018	21h 45min	50–70	7	10	3	64–88	65–81
8	0.55/0.55	14.11.2018	22h 00min	50–70	8	4	0	65–82	0–0
8	0.55/0.60	14.11.2018	18h 20min	50–70	8	6	0	65–86	0–0
9	0.55/0.55	27.11.2018	22h 20min	35–20	9	47	42	50–91	50–68
9	0.55/0.60	27.11.2018	23h 20min	95–45	9	8	3	62–79	58–76
10	0.55/0.55	28.11.2018	23h 20min	35–20	10	17	13	51–72	50–63
10	0.55/0.60	28.11.2018	22h 20min	50–85	10	0	0	0–0	0–0
11	0.55/0.55	29.11.2018	23h 40min	38–25	11	25	33	50–81	50–85
11	0.55/0.60	29.11.2018	26h 20min	55–45	11	27	17	53–80	54–77
12	0.55/0.55	30.11.2018	18h 05min	30–75	12	34	30	50–81	50–88
12	0.55/0.60	30.11.2018	18h 55min	45–48	12	2	6	70–80	65–77
13	0.55/0.55	01.12.2018	25h 40min	30–75	13	28	23	50–92	60–85
13	0.55/0.60	01.12.2018	26h 00min	45–48	13	6	3	61–72	67–80
14	0.55/0.55	02.12.2018	18h 05min	30–76	14	26	20	50–82	54–77
14	0.55/0.60	02.12.2018	18h 15min	45–49	14	2	7	75–75	57–79
15	0.55/0.55	03.12.2018	26h 10min	35–20	15	44	33	50–78	51–80
15	0.55/0.60	03.12.2018	28h 05min	50–85	15	20	19	61–88	55–81
16	0.55/0.55	04.12.2018	16h 00min	30–75	16	16	15	50–78	53–73
16	0.55/0.60	04.12.2018	16h 15min	45–48	16	9	12	54–85	58–84
17	0.55/0.55	06.12.2018	23h 00min	30–75	17	26	23	51–78	51–76
17	0.55/0.60	06.12.2018	23h 25min	45–48	17	61	52	59–96	55–87
18	0.55/0.55	07.12.2018	25h 20min	30–75	18	31	11	50–73	50–70
18	0.55/0.60	07.12.2018	22h 20min	45–48	18	3	11	62–75	57–77
19	0.55/0.55	08.12.2018	24h 05min	30–75	19	51	40	50–86	50–84
19	0.55/0.60	08.12.2018	27h 55min	45–48	19	20	12	53–88	61–81
20	0.55/0.55	09.12.2018	22h 50min	30–75	20	54	39	50–81	50–82
20	0.55/0.60	09.12.2018	18h 10min	45–48	20	15	9	53–77	54–85
21	0.55/0.55	10.12.2018	16h 30min	30–75	21	47	58	52–76	50–86
21	0.55/0.60	10.12.2018	16h 05min	45–48	21	22	21	50–82	55–72

The rows highlighted in grey indicates sets used in the analysis (sets containing at least 10 saithe). The setups with 0.55mm nylon PA gillnets / 0.55- or 0.60-mm biodegradable gillnets are indicated by 0.55/0.55 and 0.55/0.60.

**Table 5:** Catch rate and fit statistic results from the 0.55 and 0.60 mm biodegradable gillnets vs. the 0.55 mm nylon PA set based on valid deployments for saithe.

Length (cm)	Catch ratio (%)	
	0.55 mm	0.60 mm
50	103.33 (64.00–199.22)	126.66 (70.30–608.14)
55	94.42 (73.90–140.63)	124.11 (76.96–319.85)
60	86.58 (70.16–110.11)	110.00 (70.75–186.24)
65	80.20 (63.52–92.19)	93.93 (60.67–137.33)
70	75.54 (53.68–88.66)	79.96 (53.35–110.59)
75	72.85 (46.76–95.12)	68.32 (46.18–97.93)
80	72.49 (47.52–119.27)	57.43 (36.45–96.40)
85	75.14 (43.22–261.02)	45.23 (25.14–79.05)
90	81.86 (31.08–1550.13)	32.05 (8.66–67.15)
95	93.83 (19.72–8043.05)	23.18 (1.29–62.48)
Average	83.40 (71.34–94.86)	83.87 (66.36–104.92)
P–value	0.6438	0.4114
Deviance	33.29	35.19
DOF	37	34

Values in parentheses represent the 95% confidence intervals. DOF denotes the degrees of freedom.

**Fig 6:**
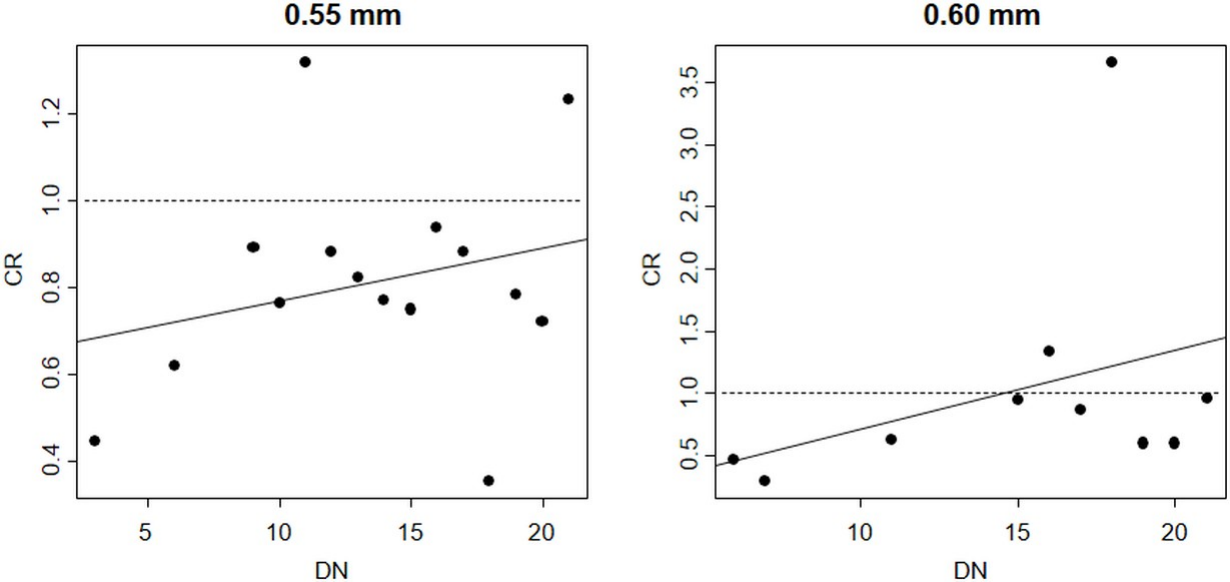
Fit of the linear model (thick solid line) testing the effect of number of times the gillnets were deployed (DN) on *CR*_*average*_ saithe. At 1.0, both biodegradable and nylon PA gillnets fish equally. The circle marks represent the experimental length-integrated catch ratio (*CR*_*average*_) for individual deployments.

No clear effect of the number of times the gillnets were deployed on the relative catch efficiency between biodegradable and nylon PA gillnet was found for saithe (Fig 6). For gillnets of 0.55 and 0.60 mm an increase was indicated. However, in neither case the effect was found to be significant as the p-value for the slope parameter α > 0.05 (Table 6).

**Table 6:** Results from linear modelling of the effect of number of times the gillnets were deployed on *CR*_*average*_ for saithe.

	parameter	value	Standard error	Significance (*p-*value)
	*α*	0.01205	0.01286	0.36593
0.55 mm	*β*	0.64828	0.18646	0.00409
	*R*^*2*^*-value*	0.06324		
	*α*	0.06365	0.06074	0.325
0.60 mm	*β*	0.08237	0.96074	0.934
	*R*^*2*^*-value*	0.1207		

**Fig 7:**
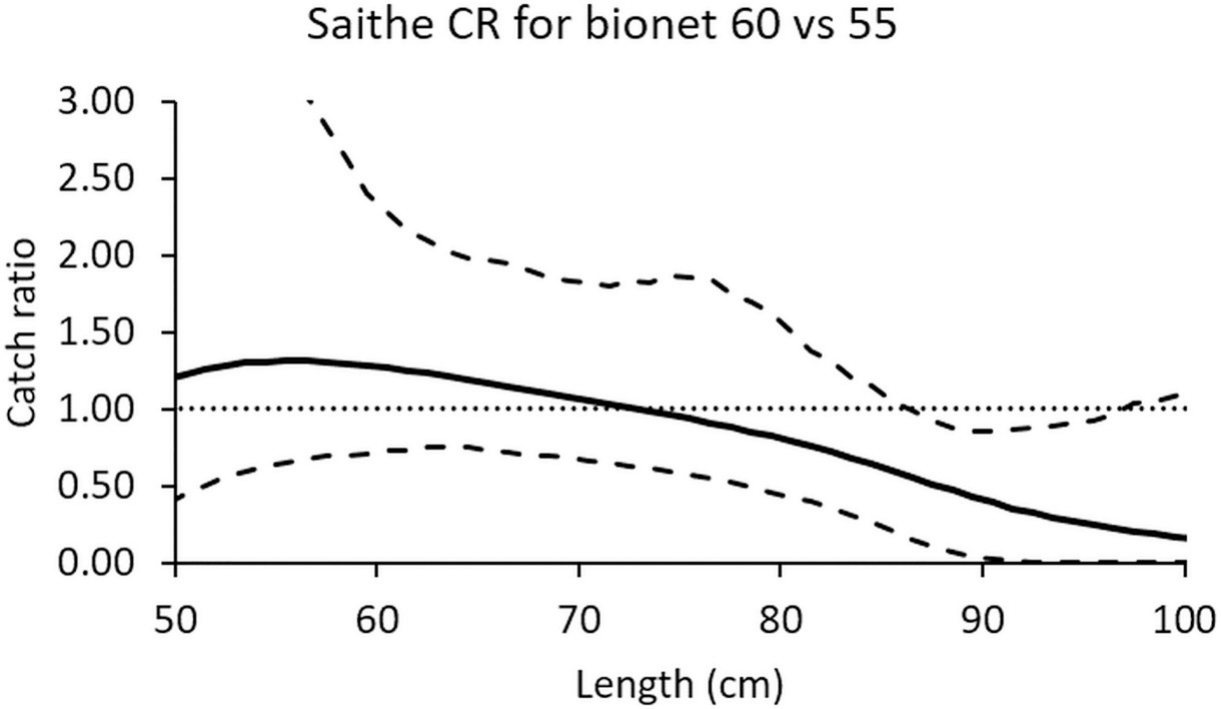
Relative catch efficiency between the two biodegradable gillnet designs for saithe (solid line). The dashed curves represent the 95% confidence interval of the estimated catch ratio curve. The dotted line at 1.0 indicates the baseline where fishing efficiency of both gillnet types is equal.

Increasing the monofilament diameter from 0.55 mm to 0.60 mm did not have a significant effect on the catch efficiency of biodegradable gillnets, except for the length classes between 88 and 97 cm. At these length classes the 0.55 mm gillnet caught significantly more saithe than the 0.60 mm nets (Fig 7).

### Mechanical properties of the gillnets

New 0.55 mm nylon PA gillnets were 9.7% (t-test, p = 2.5×10^−5^) stronger than 0.55 mm biodegradable gillnets, and as strong as the 0.60 mm biodegradable gillnets (t-test, p = 0.402). New 0.55 mm nylon PA gillnets elongated significantly less at break than the 0.55 mm (17.0%; t-test, p = 7.1× 10^−17^) and 0.60 mm (16.6%; t-test, p = 1.6×10^−19^) biodegradable gillnets. The *k*_*1*_ and *k*_*2*_ of new nylon PA nets were significantly higher (t-test, p < 0.001) than the new 0.55 mm and 0.60 mm gillnets (Table 7).

**Table 7:** Mechanical properties of the gillnets.

	Tensile strength (kg)			Elongation at break (%)			*k*_*1*_			*k*_*2*_		
Gillnet type	New	Used	% difference	New	Used	% difference	New	Used	% difference	New	Used	% difference
0.55mm Nylon PA	14.6 (14.2–15.1)	14.6 (13.9–15.1)	–0.0	32.7 (31.9–33.4)	27.9 (26.9–28.9)	–14.6	0.2857 (0.2808–0.2906)	0.3437 (0.3382–0.3492)	20.3%	0.4131 (0.3994–0.4268)	0.4709 (0.4644–0.4773)	13.9%
0.55mm Biodegradable	13.3 (13.1–13.5)	11.5 (10.9–12.1)	–13.5	39.4 (38.8–39.9)	37.8 (36.6–39.1)	–4.0	0.2078 (0.2027–0.2130)	0.2319 (0.2280–0.2358)	11.6%	0.2469 (0.2406–0.2532)	0.2511 (0.2386–0.2637)	1.7%
0.60mm Biodegradable	14.9 (14.5–15.3)	12.4 (11.7–13.0)	–16.7	39.2 (38.5–39.8)	37.9 (36.3–39.4)	–8.1	0.2619 (0.2571–0.2666)	0.2714 (0.2629–0.2799)	3.6%	0.3074 (0.2991–0.3158)	0.3227 (0.3112–0.3342)	4.9%

Mean tensile strength, elongation at break, *k*_*1*_ and *k*_*2*_ with 95% confidence intervals (in brackets) for new and used gillnets.

Used 0.55 mm nylon PA gillnets were significantly stronger (26.9%; t-test, p = 1.7× 10^−8^) and (17.7%; t-test, p = 2.2×10^−5^) than 0.55mm and 0.60mm biodegradable gillnets, respectively. Used 0.55 mm nylon PA gillnets elongated significantly less (26.2%; t-test, p = 4×10^−14^) and (26.4%; t-test, p = 8.2×10^−12^) at break than 0.55 mm and 0.60 mm used biodegradable gillnets, respectively. The *k*_*1*_ and *k*_*2*_ of used nylon PA gillnets was significantly higher (t-test, p < 0.001) than that for 0.55 mm and 0.60 mm used biodegradable gillnets, respectively (Table 7).

Nylon PA gillnets were as strong, elongated 14.6% less at break (from 32.7 to 27.9%; t-test, p = 1.49× 10^−8^), and were significantly more elastic (*k*_*1*_ = 20.3% and *k*_2_ = 13.9%; t-test, p < 0.001) after having been deployed 21 times at sea. Both types of biodegradable gillnets suffered significant reductions in tensile strength (t-test, p < 0.001). The tensile strength of the 0.55 mm biodegradable gillnet decreased from 13.3 to 11.5 kg and that of the 0.60 mm biodegradable gillnet decreased from 14.9 to 12.4 kg after being used 21 times at sea. The 0.55 and 0.60 mm nets elongated significantly less at break (4.0%; t-test, p = 3.31× 10^−2^) and (8.1%; t-test, p = 7.70×10^−4^), respectively, and *k*_*1*_ and k_*2*_ increased after use (Table 7).

**Fig 8:**
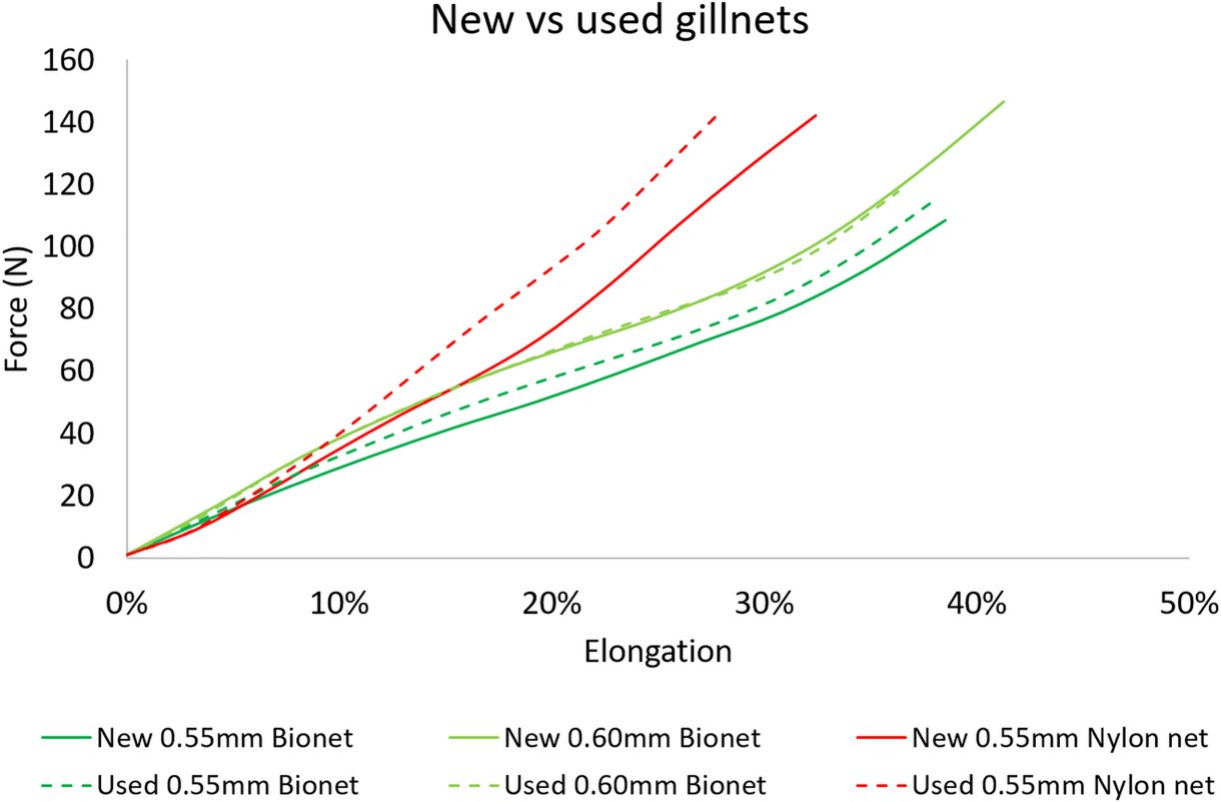
Force–elongation curves of new and used gillnets. Elongation is shown as a percentage relative to the initial length.

The fitted force-elongation curves from tensile testing (Fig 8) shows that used nylon PA gillnets exhibited an increase in stiffness, while used biodegradable gillnets experienced a slight decrease.

## Discussion

Increasing the monofilament thickness of biodegradable gillnets from 0.55 to 0.60 mm to match the tensile strength of the 0.55 mm nylon PA gillnets did not improve their catch efficiency. No difference in breaking strength between 0.55 mm nylon PA and 0.60 mm biodegradable gillnets was detected when the gillnets were new. However, the 0.55 mm nylon PA gillnets caught significantly more cod and saithe than the 0.60 mm biodegradable gillnets during the fishing season and generally showed better catch rates for most length classes. Increasing the monofilament thickness of biodegradable gillnets from 0.55 to 0.60 mm had no effect on the catch efficiency of cod, but it had a significant effect on large saithe (87–97 cm). Our results are consistent with those reported by Grimaldo et al. [18, 19] for the catch characteristics of gillnets for cod, saithe and Greenland halibut (*Reinhardtius hippoglossoides*), those of Bae et al. [24] for flounder (*Cleisthenes pinetorum*), and those of Kim et al. for yellow croaker (*Larimichthys polyactis*). These researchers found that the fishing efficiency of nylon PA gillnets was 1.1- to 1.4-times higher than biodegradable gillnets and concluded that differences in the mechanical properties of the materials (i.e., tensile strength) could explain the differences in catch efficiency. All of these studies showed that biodegradable gillnets were generally 10–16% weaker and elongate 8–10% more at break than nylon PA gillnets of similar twine diameter. However, none of these studies carried out a more comprehensive assessment of the potential effects of other mechanical properties (i.e., strength, elongation, elasticity, stiffness) on the catch efficiency of the gillnets. The results of our study suggests that tensile strength may not be the main cause of the low catch efficiency of biodegradable gillnets relative to that of nylon PA gillnets, and we therefore speculate whether the elasticity and stiffness may better explain the catch efficiency patterns of nylon PA and biodegradable gillnets.

**Fig 9:**
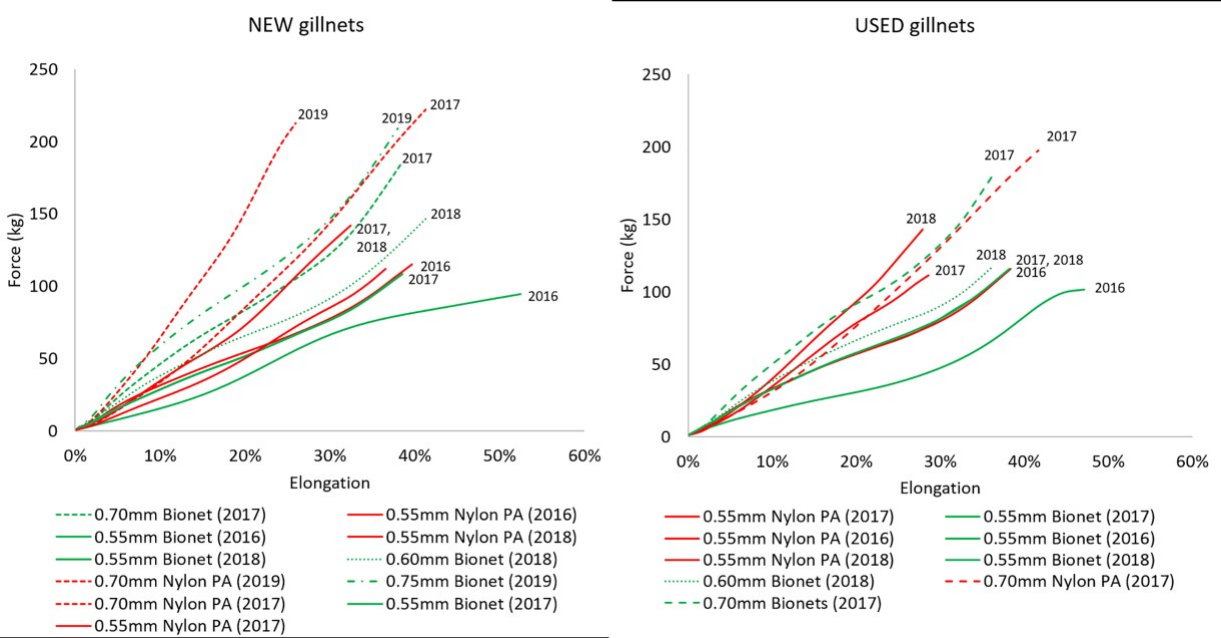
Force-elongation curves of new and used gillnets from experiments carried out in 2016–2019. Elongation is given as a percentage relative to the initial length.

Significant differences in the elasticity and stiffness were found between new biodegradable and nylon PA gillnets and therefore these two parameters may have caused the differences in catch efficiency between the gillnets. Tensile testing (Fig 8) shows an increase in the stiffness of used nylon PA monofilaments, while the used biodegradable monofilaments experienced the opposite effect. The increased stiffness of monofilaments may indicate degradation (or deterioration) of the polymer material. Based on these results, we speculate whether the biodegradable gillnets became too elastic and consequently fish could easily press themselves through the meshes of the gillnet and avoid capture. The force-elongation curves from earlier experiments obtained from biodegradable and nylon PA gillnet samples (Fig 9) give an indication of the differences in elongation and stiffness between these two types of gillnets. Although Fig 9 shows a large variation in the results for type of gillnets and year, it is possible to see a tendency for the nylon PA gillnets to be stiffer than the biodegradable gillnets, both when new and used. It also seems that used biodegradable gillnets tend to become less stiff and elongate less than nylon PA gillnets after use.

The large variability observed in the force-elongation curves from the biodegradable and nylon PA nets used between 2016 and 2019 (Fig 9) may be partially explained by the fact that gillnets sets were individually ordered for the specific fishing experiments in those years and there is a chance that the nets did not have the same mechanical properties. As shown by Kim et al. [39], biodegradable nets have a low melting point (114°C) and are therefore difficult to be heat-treated. Consequently, the performance of biodegradable nets varies depending on the manufacturer and the heat treatment of the nets that could vary between 55 to 75°C [39].

The elasticity and stiffness of nylon PA and biodegradable materials are probably closely related to the way these two types of gillnet catch fish, better known as "catching modes" [40]. For instance, a stiffer and less elastic material may catch more fish by gilling, while a more flexible and elastic material can fish more by snagging. A quantification of the number and length distribution of fish caught per catching mode type can potentially provide information on the effect that elasticity and stiffness have on the catch efficiency of gillnets. This information can also be used for improving size selectivity and to narrow the wide selection range that traditional gillnets are known for. Knowing more about the effect of elasticity and stiffness on the caching modes can also lead to the enhancement of some catch methods to improve catch quality, since wedging and entangling are known to cause marks in the fish and reduce the quality of the filet, while snagging and gilling may yield better quality fish. Unfortunately, our experimental setups did not allow us to investigate how the material elasticity affects the catch efficiency of the gillnets, and consequently this is only a hypothesis that should be investigated in future experiments.

The deterioration of nylon PA and biodegradable gillnets in this experiment was the result of chemical and mechanical changes that occurred during the three-month experimental period. Different mechanisms of degradation may have acted simultaneously on the nylon PA and biodegradable fibers, and some probably had a stronger effect than others. Although this experiment was unable to identify and quantify the effect of specific mechanisms of degradation of the gillnets that were studied, possible degradation mechanisms during the field experiments are microbiological degradation, hydrolysis, oxidation, and mechanical damage (i.e., abrasion in the hauling machine, friction due to contact with hard surfaces when the gillnets were operated on deck). Polymers are also known to be vulnerable to UV-exposure, however since the experiment was carried out during the first part of the polar night period in northern Norway, we consider the effect of UV-radiation to be negligible.

## Supporting information

S1 File Catch data for individual sets for cod. The catch data consists of count data for numbers of cod caught in the biodegradable gillnets (Test 1) and nylon PA gillnets (Test 2) for each size class (Length) corresponding to total fish length.
(ZIP)

S2 File Catch data for individual sets for saithe. The catch data consists of count data for numbers of saithe caught in the biodegradable gillnets (Test 1) and nylon PA gillnets (Test 2) for each size class (Length) corresponding to total fish length.
(ZIP)
